# Identification of an emerging cucumber virus in Taiwan using Oxford nanopore sequencing technology

**DOI:** 10.1186/s13007-022-00976-x

**Published:** 2022-12-22

**Authors:** Zi-Xuan Dong, Chian-Chi Lin, Yuh-Kun Chen, Chia-Cheng Chou, Tsung-Chi Chen

**Affiliations:** 1 Department of Medical Laboratory Science and Biotechnology, Asia University, Wufeng, Taichung, Taiwan; 2 Department of Plant Pathology, National Chung Hsing University, Taichung, Taiwan; 3 National Laboratory Animal Center, National Applied Research Laboratories, Taipei, Taiwan

**Keywords:** Cucumber Bulgarian latent virus (CBLV), *Tombusvirus*, Nanopore sequencing, *Cucumis sativus*, Virus detection

## Abstract

**Background:**

In June 2020, severe symptoms of leaf mosaic and fruit malformation were observed on greenhouse-grown cucumber plants in Xizhou Township of Changhua County, Taiwan. An unknown virus, designated CX-2, was isolated from a diseased cucumber sample by single lesion isolation on *Chenopodium quinoa* leaves. Identification of CX-2 was performed. Moreover, the incidence of cucumber viruses in Taiwan was also investigated.

**Methods:**

Transmission electron microscopy was performed to examine virion morphology. The portable MinION sequencer released by Oxford Nanopore Technologies was used to detect viral sequences in dsRNA of CX-2-infected leaf tissue. The whole genome sequence of CX-2 was completed by Sanger sequencing and analyzed. Reverse transcription-polymerase chain reaction (RT-PCR) with species-specific primers and indirect enzyme-linked immunosorbent assay (ELISA) with anti-coat protein antisera were developed for virus detection in the field [see Additional file 1].

**Results:**

Icosahedral particles about 30 nm in diameter were observed in the crud leaf sap of CX-2-infected *C. quinoa* plant. The complete genome sequence of CX-2 was determined as 4577 nt long and shared 97.0–97.2% of nucleotide identity with that of two cucumber Bulgarian latent virus (CBLV) isolates in Iran and Bulgaria. Therefore, CX-2 was renamed CBLV-TW. In 2020–2022 field surveys, melon yellow spot virus (MYSV) had the highest detection rate of 74.7%, followed by cucurbit chlorotic yellows virus (CCYV) (32.0%), papaya ringspot virus virus watermelon type (PRSV-W) (10.7%), squash leaf curl Philippines virus (SLCuPV) (9.3%), CBLV (8.0%) and watermelon silver mottle virus (WSMoV) (4.0%). Co-infection of CBLV and MYSV could be detected in field cucumbers.

**Conclusion:**

The emerging CBLV-TW was identified by nanopore sequencing. Whole genome sequence analysis revealed that CBLV-TW is closely related, but phylogenetically distinct, to two known CBLV isolates in Bulgaria and Iran. Detection methods including RT-PCR and indirect ELISA have been developed to detect CBLV and to investigate cucumber viruses in central Taiwan. The 2020–2022 field survey results showed that MYSV and CCYV were the main threats to cucumbers, with CBLV, SLCuPV and WSMoV were occasionally occurring.

**Supplementary Information:**

The online version contains supplementary material available at 10.1186/s13007-022-00976-x.

## Background

Cucurbitaceae is a large plant family with economically important crops, of which the fruits are used for nutritional and medicinal purposes. Cucumbers (*Cucumis sativus* L.), melons (*Cucumis melo* L.), watermelons (*Citrullus lanatus* (Thunb.) Matsum. and Nakai), and pumpkins and squashes (*Cucurbita* spp.) are the major cucurbit species as important vegetable crops and cultivated worldwide [1]. According to the statistics of Food and Agriculture Organization of the United Nations (http://www.fao.org/faostat/en/#data/QC), global productions of the major cucurbit crops were near 450 megatonnes (Mt) in 2020. Cucurbit crops are also economically important in Taiwan with the planting area of more than 20,000 ha and an annual output value of about US$ 250 million (Agriculture and Food Agency, Council of Agriculture, Executive Yuan, 2020, https://agrstat.coa.gov.tw/sdweb/public/inquiry/InquireAdvance.aspx). Cultivation of cucurbit crops is always challenged by pathogenic microorganisms, especially viruses [2]. Numerous viruses infecting cucurbit crops have been reported around the world, of which squash leaf curl Philippines virus (SLCuPV) of the genus *Begomovirus* [3], cucurbit chlorotic yellows virus (CCYV) of the genus *Crinivirus* [4], zucchini yellow mosaic virus (ZYMV) [5] and papaya ringspot virus watermelon type (PRSV-W) [6] of the genus *Potyvirus*, and melon yellow spot virus (MYSV) [7] and watermelon silver mottle virus (WSMoV) [8] of the genus *Orthotospovirus* are the most prevalent viruses in Taiwan in the last decade. The viruses can be efficiently spread by tiny insects, CCYV and SLCuPV are transmitted by whiteflies; ZYMV and PRSV-W are transmitted by aphids; and MYSV and WSMoV are transmitted by thrips, resulting in a significant decrease in cucurbit fruit yield and quality [9].

Diagnosis of viral disease with symptomatology is difficult because the symptoms caused by viral infections is similar to nutritional deficiencies. Virus identification is critical for crop disease management. Although various serological and molecular detection methods have been developed to identify virus species, the exploration of unknown viruses without *a priori* knowledge by these methods is still challenged. Fortunately, this issue can be solved with high-throughput sequencing (HTS) technologies coupled with metagenomic analysis [10]. Several HTS platforms have been launched, such as Roche 454, Illumina, SOLiD, PacBio and Nanopore. Due to the highest accuracy (> 99.9%), biggest output and lowest cost, the Illumina sequencing platforms become the most commonly used tool in plant virology research, including virus detection and whole genome sequencing. However, hyper-efficient computer instruments are required to process the enormous output data of hundreds of gigabases consisting of short 200-bp reads for *de novo* assembly and sequence alignment [11]. MinION, released by Oxford Nanopore Technologies (ONT, Oxford, UK), is a portable single-molecule sequencer that was designed for researchers with limited resources, and has become an efficient tool for plant virus diagnosis and identification [12–15]. MinION using nanopore technology can real-time analyze the complete sequence of a single nucleic acid molecule by pulling a single nucleic acid strand through a biologic nanopore, anchored on to it by a molecular motor protein, and determining the nucleotide (nt) sequence by measuring voltage changes [16]. Although MinION is convenient and cost-effective, the accuracy of base calling is relatively low, ranging from 65 to 88% [16, 17]. Nevertheless, consensus sequences obtained by *de novo* assembly or mapping to a reference can be comparable to Illumina sequencing [18].

In June 2020, cucumbers cultivated in a greenhouse in Xizhou Township of Changhua County, Taiwan suffered from severe mosaic disease and deformed fruit symptoms. Symptomatic cucumber samples were collected for virus detection and identification. The virus isolate, designated CX-2, was isolated from one of the collected samples through three successive single-lesion isolations on *Chenopodium quinoa* leaves. CX-2-inoculated *C. quinoa* leaf tissue was negative for CCYV, SLCuPV, ZYMV, PRSV-W, MYSV and WSMoV. Icosahedral particles about 30 nm in diameter could be observed in the crud leaf sap. Nanopore sequencing performed with MinION indicated CX-2 as cucumber Bulgarian latent virus (CBLV). Subsequently, the whole genome sequence of CX-2 was verified by Sanger sequencing to demonstrate the identity of the virus. The current incidence of CBLV and other viruses in cucumber crops in Taiwan is also addressed in this study.

## Materials and methods

### Virus source and inoculation

**Fig. 1:**
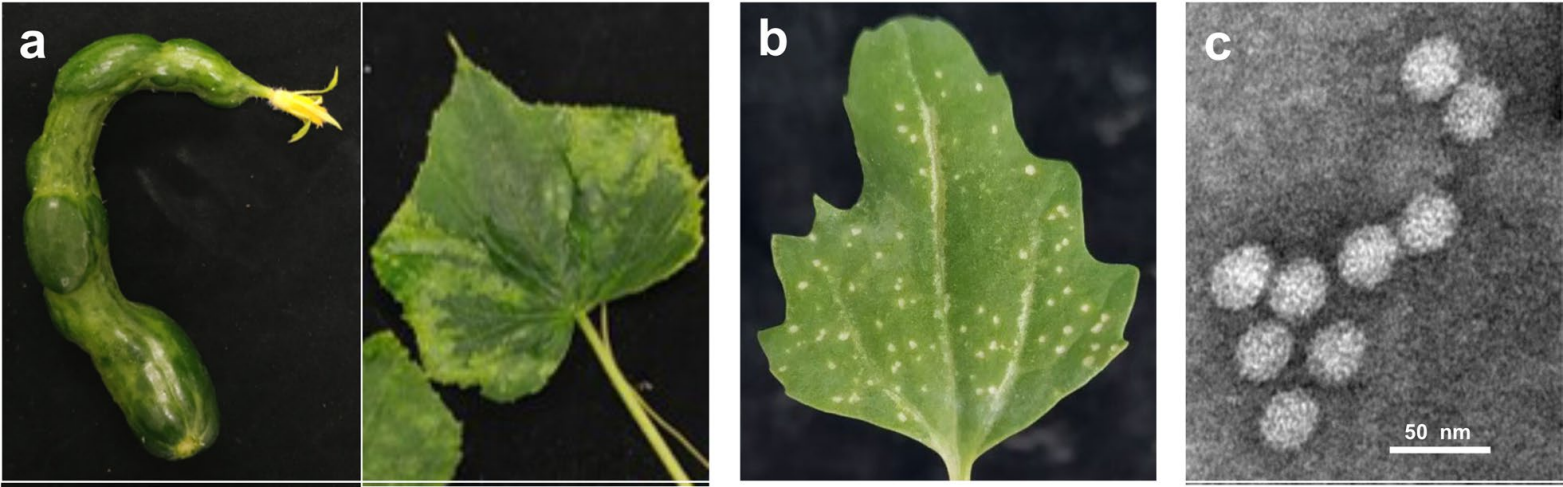
Observation of host effects and virion morphology of the emerging tombusvirus-like virus. **a** Symptoms showing leaf mosaic (right) and fruit malformation (left) were observed on field cucumber plants in Xizhou Township of Changhua County, Taiwan in June 2020. **b** The virus isolate CX-2, isolated from the diseased cucumber sample ‘2106-2’ through three successive single lesion transfers on C*henopodium quinoa* leaves, induces necrotic spots on inoculated *C. quinoa* leaves 3 days post-inoculation (3 dpi). **c** Negative-stained icosahedral virus particles of about 30 nm in diameter were observed in the crude sap of CX-2-infected *C. quinoa* leaves 3 dpi

Diseased cucumbers showing severe symptoms of leaf mosaic and fruit malformation (Fig. 1a) were collected in Xizhou Township of Changhua County, Taiwan in June 2020. The virus isolate CX-2 was isolated from the diseased cucumber sample ‘2106-2’ through three successive single lesion transfers on *C. quinoa* leaves. Manual mechanical inoculation was performed for virus transfer, and crude sap of virus-infected leaf tissue ground in 10 mM potassium phosphate buffer (pH 7.0) containing 10 mM sodium sulfite was used as inoculum. The virus was propagated in *C. quinoa* and *Nicotiana benthamiana* plants under greenhouse conditions for future studies.

### Transmission electron microscopy (TEM)

Small pieces (5 × 5 mm^2^) of *C. quinoa* leaves inoculated with viruses were ground and sap droplets were mixed with 2% glutaraldehyde for fixation. Copper grids were floated on the sample droplets for 1 min, the residual liquid on the copper grids was removed with filter paper, and then stained with 2% uranyl acetate. A JEM-2000EX transmission electron microscope (JEOL Ltd., Japan) was used for examination.

### Viral dsRNA extraction

Double-stranded RNA was extracted from virus-inoculated *C. quinoa* leaf tissue as previously described [19]. Briefly, 200 mg of fresh leaf tissue ground with liquid nitrogen was immediately suspended in 600 µl of EBA-30% E buffer (50 mM Tris-HCl pH 8.5, 50 mM EDTA, 3% SDS, 1% β-mercaptoethanol, 1% PVPP-40, adjusted to 30% ethanol) by rolling for 20 min at room temperature and then centrifuged at 16,110 *g* for 15 min at 4 °C. The supernatant was collected and adjusted to a final concentration of 20% ethanol and then loaded to a micro-column, in which 600 µl of cellulose CF-11 (Whatman, Buckinghamshire, UK) had been equilibrated with 1× STE-20% E buffer (10 mM Tris-HCl pH 8, 100 mM NaCl, 1 mM EDTA, pH 8.0, adjusted to pH 7.8 and 20% ethanol). The micro-column was centrifuged at 100 *g* for 2 min to remove liquid and then washed twice by adding 450 µl of 1× STE-20% E buffer and centrifuging at 100 *g* for 2 min. The dsRNA was eluted from the column by adding 400 µl of 1× STE buffer twice and centrifuging at 100 *g* for 2 min. The eluate was collected and mixed with an equal volume of isopropanol, rolled for 10 min at room temperature and centrifuged at 16,110 *g* for 30 min at 4 °C. The dsRNA pellet was washed with 70% ethanol, air-dried at room temperature, and dissolved in 50 µl of RNase-free water.

### cDNA library construction and ONT nanopore sequencing

First strand cDNA was synthesized with SuperScript IV reverse transcriptase (ThermoFisher Scientific, Waltham, MA) and random hexamers starting from 200 ng of dsRNA. The second strand DNA was synthesized with Klenow fragment of DNA polymerase I (New England Biolabs, Ipswich, MA). The synthesized dsDNA was precipitated by 100% ethanol and then end-repaired and A-tailed by adding with the EA enzyme provided by the KAPA Hyper Prep kit (KAPA Biosystems, Wilmington, MA). The ligation of the treated dsDNA with adaptor motor mix was performed by the ONT ligation sequencing kit following the ONT protocol SQK-LSK109. The prepared dsDNA was loaded on flow cells and sequencing was performed with MinION for eight hours. Reads obtained from sequencing were real-time analyzed using the ONT EPI2ME WIMP workflow.

### Metagenomic analysis for taxonomic classification

The taxonomic classification of sequence data was performed by Kraken 2 [20]. The non-redundant nt database was downloaded from the GenBank of National Center for Biotechnology Information (NCBI) and used for building a classification database for Kraken 2 (*k* = 35, ℓ = 31). Dustmasker and segmasker programs [21] provided as part of NCBI’s BLAST suite were used to mask low-complexity regions. Bracken was used to estimate abundance at standard taxonomy level [22]. The output results were confirmed by using BLASTn in NCBI with customized Python scripts.

### Viral genome sequencing by Sanger sequencing

Total RNA was extracted from virus-infected *C. quinoa* leaf tissue using the Plant Total RNA Miniprep Purification kit (GeneMark, GMbiolab, Taichung, Taiwan) according to the manufacturer’s instructions. The nt sequences of primers used to amplify viral genome fragments in reverse transcription-polymerase chain reaction (RT-PCR) are shown in  (see Additional file 2: Table S1). RT-PCR was performed according to the instructions of the One-Step RT-PCR kit (GeneMark), 2 µl of total RNA (1 µg), 12.5 µl of 2× RT buffer (dNTPs, Mg2^+^ and enzyme stabilizer), 0.5 µl of enzyme mix (reverse transcriptase and *Taq* DNA polymerase), 0.5 µl forward and reverse primers (200 nM for final concentration) and 0.1 µl of RNase block were mixed in a final volume of 25 µl. First strand cDNA was synthesized at 50 °C for 30 min, followed by inactivation of reverse transcriptase and activation of *Taq* DNA polymerase at 94 °C for 2 min. PCR was performed for 35 cycles of 30 s at 94 °C, 30 s at 58 °C, and 1 min at 72 °C and an additional final reaction at 72 °C for 7 min. PCR products were analyzed by 1% agarose gel electrophoresis and then eluted with the Micro-Elute DNA Clean/Extraction kit (GeneMark) following the manufacturer’s instructions. The amplicons were cloned by TOPO TA cloning (Invitrogen), ligated with the pCR2.1-TOPO vector and transformed into *E. coli* strain DH5α competent cells, according to standard protocols recommended by the manufacturer. Recombinant plasmids purified from the resulting clones were sequenced by an ABI3730XL DNA Analyzer (Perkin-Elmer Applied Biosystems, Foster City, CA) performed by Mission Biotech Company (Taipei, Taiwan). Three clones of each fragment were selected for sequencing.

### 5´ and 3´ rapid amplification of cDNA ends (RACE)

**Fig. 2:**
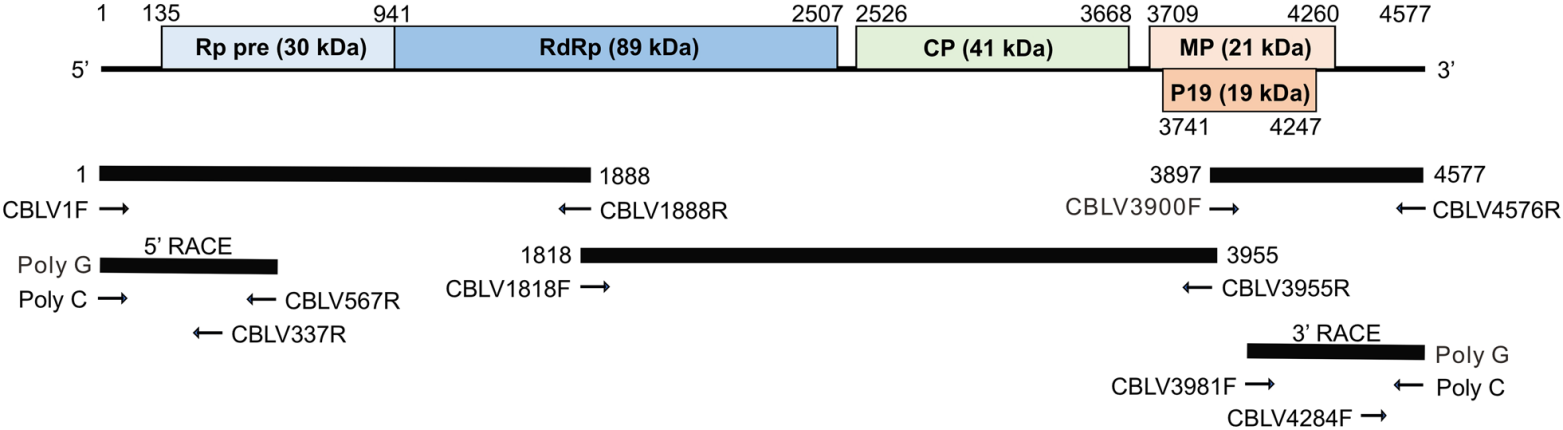
Schematic representation of genome organization of the Taiwan cucumber Bulgarian latent virus isolate (CBLV-TW). The open reading frames (ORFs) are indicated by boxes and the noncoding regions are drawn by lines. The nucleotide (nt) positions of individual ORFs and the molecular weights of viral proteins are shown. cDNA fragments amplified by RT-PCR are indicated by bold lines. Primers used for RT-PCR amplification are represented by arrows. The nt sequences of primers are listed in (see Additional file 2: Table S1)

The 5´- and 3´-ends of viral genome were confirmed by RACE [23]. Specific primers were designed from the determined nt sequences as shown in  (see Additional file 2: Table S1). Total RNA used as template was denatured at 70 °C for 10 min and then put on ice for 1 min. First strand cDNA was synthesized by SuperScript IV reverse transcriptase (Invitrogen) mixing with 200 nM of each primer at 50 °C for 60 min, followed by stop reaction at 70 °C for 15 min. After the removal of template RNA by RNase H (Invitrogen), the cDNA products were precipitated by adding 1/10 volume of 3 M sodium acetate (pH 5.2) and 2.5 volume of absolute ethanol at − 20 °C for overnight. After centrifugation at 17,000 *g* for 15 min, the pellet was resuspended in 20 µl DEPC-treated water. Subsequently, 200 nM of PolyG oligonucleotide [24] was tailed at the 3´ end of cDNA fragments by 20 U terminal deoxynucleotidy1 transferase (TdT) (New England Biolabs) at 37 °C for 30 min and the reaction was terminated at 70 °C for 10 min. The tailed cDNA fragments were mixed with 2.5 U Blend Taq-Plus (TOYOBO, Osaka, Japan), 200 nM PolyC [24] complementary to the PolyG tail and 200 nM another proper primer as shown in  [see Additional file 2: Table S1] and Fig. 2. The PCR amplification was performed for 35 cycles of 30 s at 94 °C, 30 s at 58 °C, and 1 min at 72 °C and an additional final reaction of 7 min at 72 °C. The amplified DNA fragments were cloned by TOPO TA cloning (Invitrogen) for sequencing as mentioned above.

### Viral genome sequence analysis

Genome sequences of different species of the genus *Tombusvirus*, including distinct CBLV isolates, were obtained from the GenBank database (http://www.ncbi.nlm.nih.gov/) as shown in (see Additional file 3: Table S2). Sequence identity analysis was performed by AlignX in Vector NTI Suite 10 (Invitrogen). Multiple sequence alignments were performed using the ClusalX 2.1 program in MEGA X [25]. Phylogenetic analyses were analyzed by the Neighbor-Joining method with 1000 bootstrap replicates using the Tree Explorer program in MEGA X.

### Purification of viral coat protein (CP)

CX-2 CP was purified using the ultra-speed centrifugation method previously described [26] with modifications. Briefly, 100 g of CX-2-infected *C. quinoa* leaves harvested 3 days post-inoculation (dpi) were homogenized in 300 ml of TB buffer (10 mM Tris-HCl, pH 8.0, containing 10 mM sodium sulfite and 0.1% cysteine) in a blender and centrifugated at 10,000 rpm for 15 min (GRF-L-m2.0-30, Gyrozen, Korea). The supernatants were collected and treated with 1% Triton X-100 at 4 °C for 30 min, followed by centrifugation at 25,000 rpm in Beckman Type 45 Ti rotor for 2.5 h in 20% sucrose cushion. The pellets were then resuspended in TBG buffer (TB buffer containing 10 mM glycine) for isopycnic centrifugation through 32% cesium sulfate at 35,000 rpm in Beckman SW 41 rotor for 17 h. The opalescent zones were collected and precipitated by centrifugation at 45,000 rpm in Beckman Type 70 Ti rotor for 1 h. The pellets were resuspended in TBG buffer and treated with protein sample buffer (50 mM Tris-HCl, pH 6.8, 2% sodium dodecyl sulfate (SDS), 12% glycerol, 0.01% bromophenol blue and 2% β-mercaptoethanol) at 100 °C for 3 min. Proteins were separated in 12% SDS-polyacrylamide gel electrophoresis (PAGE) and visualized by soaking the gels in cold 0.3 M KCl. The desired protein was cut and eluted from the gel using a Model 422 Elutro-Eluter (Bio-Rad, Hercules, CA). The yield of the purified CP was estimated by the software Spot Density of AlphaInnotech IS2000 (AlphaInnotech Corporation, San Leandro, CA) by comparison with the quantified bovine serum albumin (BSA) as previously described [27].

### Production of rabbit antiserum

One hundred microgram of the purified CP dissolved in 1 ml of PBS buffer (136 mM NaCl, 1 mM KH_2_PO_4_, 8 mM Na_2_HPO_4_‧12H_2_O, 2 mM KCl and 3 mM NaN_3_) was emulsified with an equal volume of Freund’s complete adjuvant (BioSmart, South Korea) and injected subcutaneously into a New Zealand white rabbit. One week later, the rabbit was injected weekly with 100 µg of the same immunogen in 1 ml of PBS emulsified with an equal volume of Freund’s incomplete adjuvant (BioSmart) for two weeks. Blood was collected weekly from the ear marginal veins of the rabbit for one month, starting from 1 week after the third injection. The collected blood was incubated at 37 °C for 1 h and antiserum was collected from the supernatant after centrifugation at 8100 *g* for 10 min.

### Enzyme-linked immunosorbent assay (ELISA)

Indirect ELISA was conducted as previously described with modifications [28] for antiserum titration and virus detection. Aliquots of 200 µl of purified protein or crude sap of plant tissue diluted with coating buffer (15 mM Na_2_CO_3_, 35 mM NaHCO_3_ and 3 mM NaN_3_) were loaded in each well of polystyrene microtitration plates. Sample-coated plates were incubated at 37 °C for 30 min and then washed with PBST (PBS buffer containing 0.05% Tween 20) for three times, each time for 3 min. Antiserum diluted in enzyme-conjugate buffer (PBST containing 2% PVP-40 and 0.2% ovalbumin) was loaded to the plates, 200 µl for each well. The plates were incubated at 37 °C for 30 min and then washed three times with PBST. The secondary antibody, alkaline phosphatase (AP)-conjugated affinipure goat anti-rabbit IgG (Jackson Immuno Research Laboratories, Inc., West Grove, PA) for rabbit antiserum, was diluted at a 1/5000 dilution in enzyme-conjugate buffer, and aliquots of 200 µl were loaded to each well. After incubation at 37 °C for 30 min and washing with PBST, 180 µl of color-developing solution (9.7% diethanolamine and 3 mM NaN_3_) containing 1 mg/ml of ρ-nitrophenyl phosphate disodium hexahydrate (ρ-NPP-Na) (GMbiolab) was loaded to each well for colorization. The absorbances at 405 nm (A_405_) were recorded by a Model 680 microplate reader (Bio-Rad) for 1 h after the addition of enzyme substrate.

### Immunoblotting

Plant leaf tissues were ground in protein sample buffer at a 1/50 dilution, denatured by boiling for 3 min, put on ice for 1 min and centrifuged at 16,110 *g* for 3 min. The supernatants were collected and separated in 12% SDS-PAGE, and then transferred onto nitrocellulose (NC) membranes in transfer buffer (25 mM Tris, 192 mM glycine and 20% methanol) at 120 V for 30 min. The NC membranes were washed with TSW buffer (10 mM Tris-HCl, pH 7.4, 154 mM NaCl, 0.25% gelatin, 0.1% Triton X-100 and 0.02% SDS) for 3 times, each time for 3 min, and then shaken with TSW buffer-diluted antibodies at room temperature for 30 min. After washing for 3 times with TSW buffer, each for 3 min, the NC membranes were incubated at room temperature with AP-conjugated affinipure goat anti-rabbit IgG (Jackson Immuno Research Laboratories) at a 1/5000 dilution in TSW buffer for 30 min. The NC membranes were then washed twice with TSW buffer for 3 min and rinsed with substrate buffer (100 mM Tris-HCl, pH 9.5, 100 mM NaCl and 5 mM MgCl_2_) for 3 min. Color development was conducted by adding 50 µl of 50 mg/ml NBT (nitro blue tetrazolium chloride) and 25 µl of 50 mg/ml BCIP (5-bromo-4-chloro-3-indoyl phosphate) in 7.5 ml substrate buffer. The reaction was stopped by submerging the NC membranes in water.

### Virus detection in fields

Symptomatic cucurbit samples were collected in fields of Yunlin, Changhua and Taichung in central Taiwan. Total RNA extracted from plant tissue by the Plant Total RNA Miniprep Purification kit (GeneMark) was used for RT-PCR analysis. The primer pairs CBLV3900F/CBLV4576R specific to CBLV (see Additional file 2), Crini-hsp70-f (5′-GCCATAACCATTACGGGAGA-3′)/Crini-hsp70-r (5′-CGCAGTGAAAAACCCAAACT-3′) to CCYV [4], MYSV-N-f (5′-GCCATGGCATGCATGTCTACCGTTACTAAGCTGACA-3′)/MYSV-N-r (5′-GTCTAGAGGTACCAACTTCAATGGACTTAGCTCTGGA-3′) to MYSV [29], WN2963 (5′-AATAATCGGTGCCAGTCCCCTT-3′)/WN3469c (5′-ATGTCTAACGTTAAGCAGCTCACA-3′) to WSMoV [29] and SLCuPV-AV1-74 F (5′-GCCCCTATGTTTCCCGTGCAGT-3′)/SLCuPV-AV1-760R (5′-CCGAATCATAAAATAGATCCGG-3′) to SLCuPV, designed in this study, were used for nucleic acid amplification. The primer pair *nad*5-s (5′-GATGCTTCTTGGGGCTTCTTGTT-3′)/*nad*5-as (5′-CTCCAGTCACCAACATTGGCATAA-3′) for amplifying *NADH dehydrogenase subunits* 5 (*nad*5) gene was used as plant internal control [30]. The One-Step RT-PCR kit (GeneMark) was used in RT-PCR analysis as described by the manufacturer. The amplification conditions were set as 50 °C for 30 min, followed by 94 °C for 2 min, and then 35 cycles of 30 s at 94 °C, 30 s at 58 °C, and 1 min at 72 °C and a final reaction at 72 °C for 7 min. Indirect ELISA was performed as mentioned above to detect MYSV, WSMoV, PRSV-W and ZYMV using individual antisera described previously [9].

## Results

### CX-2 particle morphology

CX-2, isolated from a diseased greenhouse-grown cucumber, induced visible necrotic spots on inoculated *C. quinoa* leaves at day 3 post-inoculation (Fig. 1b). Furthermore, a large number of negative-staining icosahedral virus particles with a diameter of about 30 nm were observed in the crude sap of CX-2-infected *C. quinoa* leaves 3 dpi (Fig. 1c); therefore, this leaf material was used for subsequent studies.

### Identification of CX-2 as CBLV

The dsRNA extracted from CX-2-infected *C. quinoa* leaves was used as template to construct a random primer-primed cDNA library for sequencing. The ONT MinION was used to read nt sequences. A total of 7408 reads were analyzed, of which 13 reads were mapped to CBLV of the *Tombusvirus* genus in the *Tombusviridae* family. The full-length genome sequence of the original CBLV isolate (acc. no. AY163842) was used as a reference to align with the classified reads by BLASTn, showing that the reads share nt identities of 83.0-95.7% with the reference sequence, with a genome coverage of 73.3% (see Additional file 4).

**Fig. 3:**
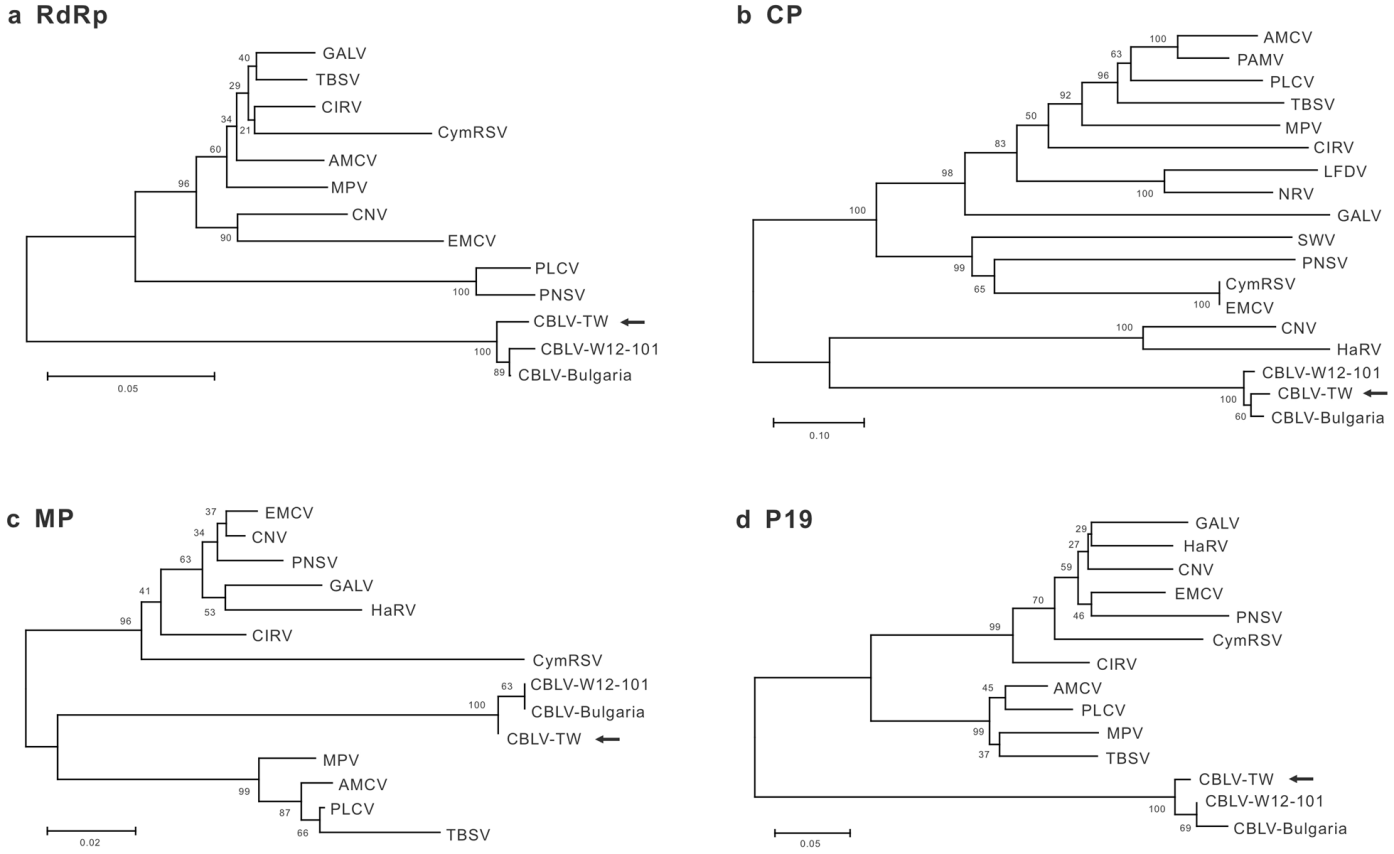
Phylogenetic relationships of CBLV-TW with other *Tombusvirus* species. **a** RNA-dependent RNA polymerase (RdRp), **b** coat protein (CP), **c** movement protein (MP) and **d** P19 are compared individually. The dendrographs were produced using the Neighbor-Joining algorithm with 1000 bootstrap replicates. CBLV-TW is indicated by arrows

Furthermore, Sanger sequencing using the newly designed primers was performed to elucidate the full-length genome sequence of CX-2. Five overlapping fragments, including 5´ and 3´ RACE, amplified from the total RNA of CX-2-infected *C. quinoa* leaves by RT-PCR were cloned for sequencing. The complete genome sequence of CX-2, containing the open reading frames (ORFs) of RNA-dependent RNA polymerase (RdRp, nt 135–2507), CP (nt 2526–3668), movement protein (MP, nt 3709–4260) and 19-kDa protein (P19, nt 3741–4247), was determined as 4577 nt in length and deposited in the GenBank (acc. no. MW359100) (Fig. 2). The whole genome sequence of CX-2 shared 97.0% and 97.2% of nt identity with those of two CBLV isolates in Iran and Bulgaria, respectively. The genomic ORFs of CX-2 shared 95.8-98.9% and 96.8-99.4% of nt and aa identities, respectively, with those of the CBLV Bulgarian and Iran (W12-101) isolates, but shared 50.5-82.3% and 33.7-83.1% of lower nt and aa identities, respectively, with those of other tombusviruses (Table 1). Phylogenetic analyses of RdRp, CP, MP and P19 indicated that CX-2 is closely related to the two known CBLV isolates (Fig. 3). Taken together, CX-2 was identified as an isolate of CBLV and renamed CBLV-TW.

**Table 1:** Nucleotide (nt) and amino acid (aa) identities (%) of genomic coding sequences of the cucumber Bulgarian latent virus Taiwan isolate (CBLV-TW) compared with those of different CBLV isolates and other tombusviruses

Virus ^a^	Genome (nt%)	RdRp		CP		MP		P19	
		nt%	aa%	nt%	aa%	nt%	aa%	nt%	aa%
CBLV-TW	100.0	100.0	100.0	100.0	100.0	100.0	100.0	100.0	100.0
CBLV-W12-101	97.0	97.0	98.4	95.8	96.8	98.7	99.4	98.6	97.0
CBLV-Bulgaria	97.2	97.2	98.1	95.9	97.3	98.9	99.4	98.6	96.4
AMCV	66.5	72.1	76.5	51.8	36.7	81.8	82.5	82.3	65.7
CIRV	66.6	72.3	76.6	51.4	34.9	81.1	83.1	81.9	65.7
CNV	67.1	72.5	76.6	53.5	38.6	78.9	81.6	78.9	61.3
CymRSV	66.1	71.8	77.0	50.8	36.1	78.6	78.8	79.0	62.2
EMCV	66.0	72.0	75.5	50.9	35.9	78.3	81.2	79.4	61.6
GALV	66.0	71.9	77.2	50.9	34.8	71.2	74.3	79.6	62.2
MPV	66.7	71.9	76.5	53.0	38.6	79.8	82.0	80.2	61.6
PLCV	65.4	67.2	70.4	50.5	35.4	81.6	82.5	82.3	64.5
PNSV	65.3	66.3	70.2	51.9	37.3	79.8	82.0	80.3	62.2
TBSV	65.7	70.8	76.9	51.2	33.7	81.1	82.0	81.5	61.6
HaRV	–^b^	–	–	52.1	36.0	78.4	80.0	79.0	60.7
LFDV	–	–	–	51.3	362	–	–	–	–
NRV	–	–	–	52.1	39.8	–	–	–	–
PAMV	–	–	–	52.4	36.8	–	–	-	–
SWV	–	–	–	52.3	36.0	–	–	–	–

^a^ See Additional file 2 for virus names and accession numbers

^b^ Represents that sequences are unavailable in the GenBank database

### Purification of CBLV-TW CP

**Fig. 4:**
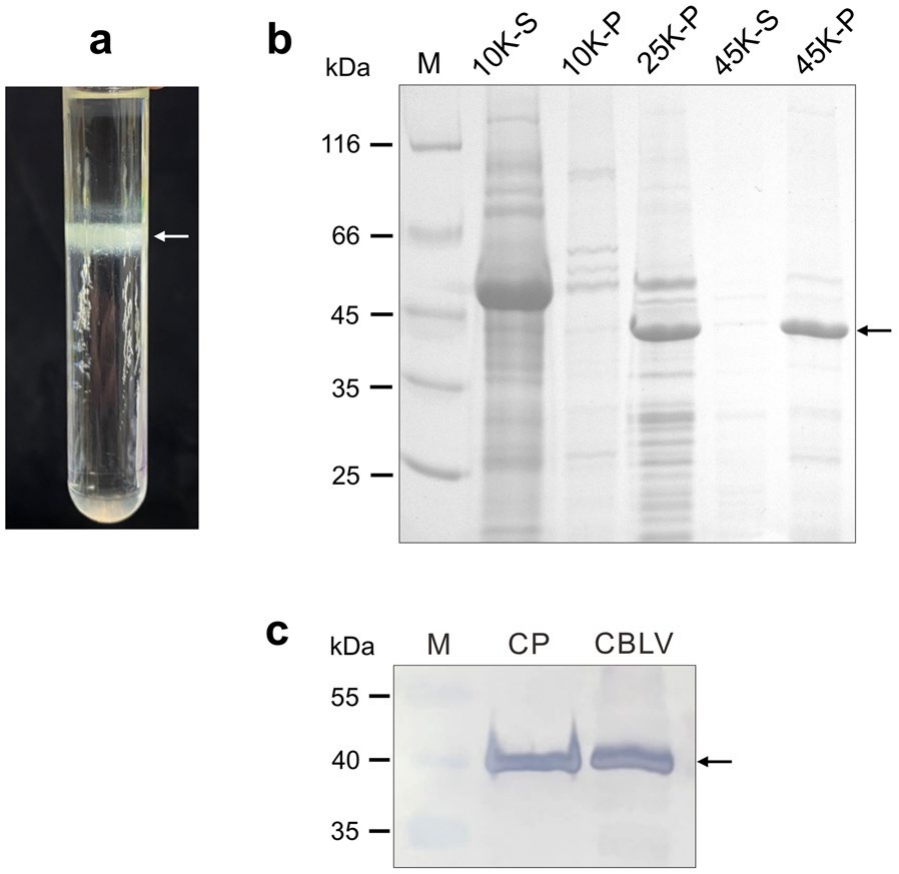
Purification of CBLV-TW coat protein (CP) from infected *Chenopodium quinoa* leaves by ultra-speed centrifugation method. **a** An obvious opalescent band indicated by a white arrow was observed after isopycnic centrifugation through 32% cesium sulfate. **b** Individual centrifugation fractions of purification procedures, 10 K rpm, 25 K rpm and 45 K rpm, were analyzed by 12% SDS-PAGE. S and P represent supernatant and pellet, respectively. **c** Immunoblotting using the produced antiserum RAs-CBLV was conducted to detect the purified CBLV CP (lane CP). The crude extract of CBLV-TW-infected *C. quinoa* leaf was used as positive control (lane CBLV). Protein marker (lane M) was loaded as molecular weight standard. CBLV-TW CP is indicated by black arrows

Leaf tissues of *C. quinoa* inoculated with CBLV-TW 3 dpi were harvested for CP purification. An obvious opalescent band was observed near the center of centrifuge tube after isopycnic centrifugation through 32% cesium sulfate. The expected 41 kDa of CP was obtained from the substances within the opalescent band (Fig. 4). Approximately 800 µg of CBLV-TW CP could be purified from 100 g of *C. quinoa* leaf tissue.

### Responses of the produced anti-CBLV CP antiserum

**Fig. 5:**
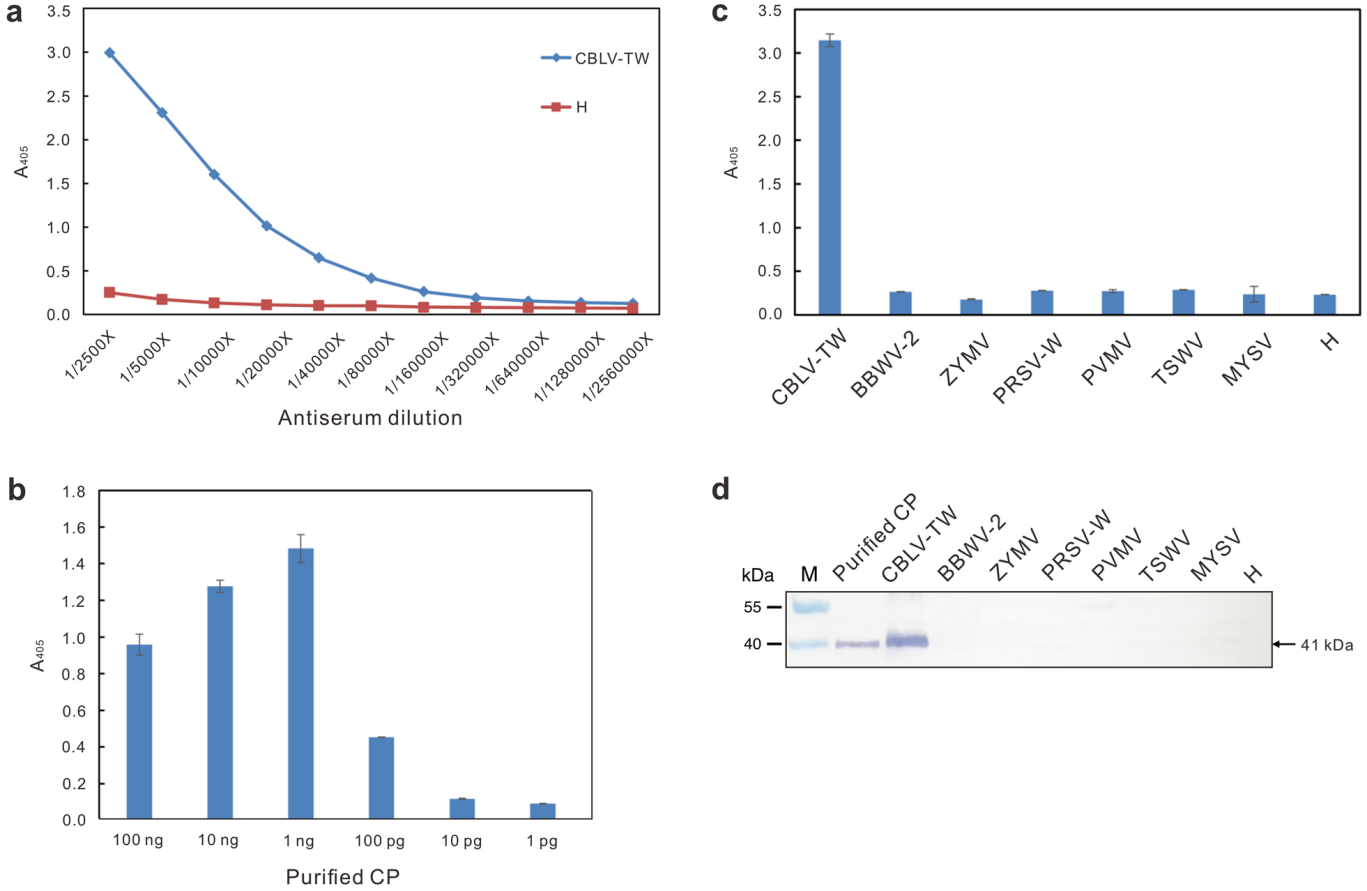
Serological assays of the produced antiserum RAs-CBLV to the CBLV-TW coat protein (CP) by indirect ELISA and immunoblotting. **a** Titration assay. The antiserum was serially diluted for assay. The crude leaf saps of CBLV-TW-infected and healthy (H) *Chenopodium quinoa* plants at a 1/50 dilution were used to determine the endpoint dilution of RAs-CBLV as 1/640,000. **b** Sensitivity assay. The quantified purified CBLV-TW CPs were used to react with RAs-CBLV at a 1/5000 dilution. The detection limit of RAs-CBLV was determined as 100 pg CP. **c **and** d** Specificity assay. Crude leaf saps of *C. quinoa* infected with BBWV-2, MYSV, PRSV-W, PVMV, TSWV and ZYMV were incubated with RAs-CBLV in indirect ELISA and immunoblotting, and no signals were detected. Purified CBLV-TW CP and CBLV-TW-infected plant tissue were used as positive controls. The expected 41-kDa CP detected in immunoblotting is indicated by an arrow

The rabbit antiserum produced from the purified CBLV-TW CP as immunogen was denoted RAs-CBLV. A serial dilution of RAs-CBLV was used to react with the crude leaf saps of CBLV-infected *C. quinoa* plants at a 1/50 dilution in indirect ELISA and the endpoint dilution was determined as 1/640,000 (the average reading of CBLV = 0.1547 compared with the healthy negative control (H) = 0.079) (Fig. 5a). RAs-CBLV was recommended to use at a 1/5000 dilution. Reciprocally, the purified CP was serially diluted to react with RAs-CBLV showing a limit of 100 pg for RAs-CBLV in indirect ELISA (Fig. 5b). RAs-CBLV was negative for other tested virus species, including broad bean wilt virus 2 (BBWV-2) of the genus *Fabavirus*, ZYMV, PRSV-W and pepper venial mottle virus (PVMV) of the genus *Potyvirus*, MYSV and tomato spotted wilt virus (TSWV) of the genus *Orthotospovirus*, as well as healthy plants in indirect ELISA (Fig. 5c) and immunoblotting (Fig. 5d).

### Detection of virus infection in field cucumber crops

**Fig. 6:**
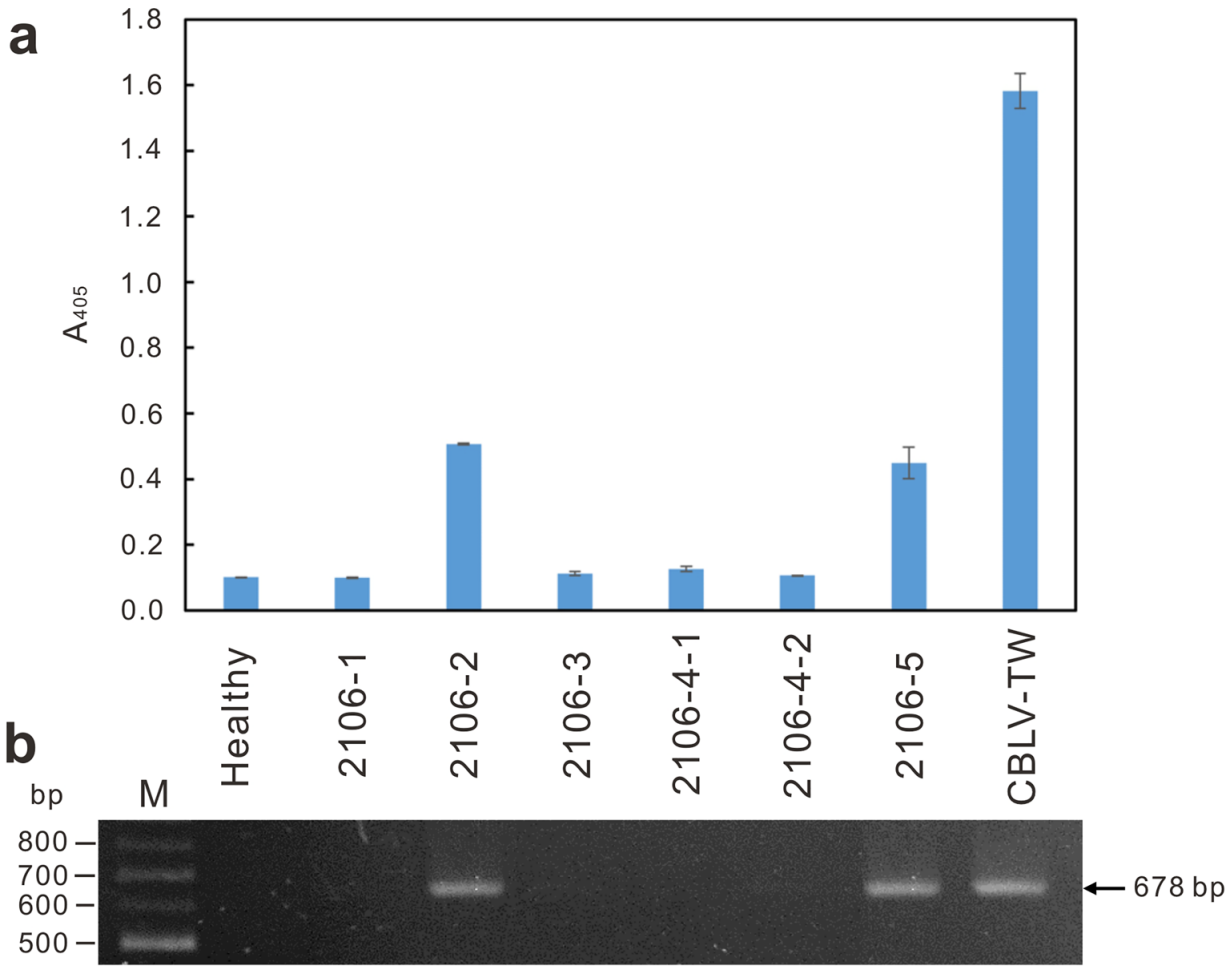
Detection of CBLV infection in field cucumber crops by indirect ELISA (**a**) and RT-PCR (**b**). The produced RAs-CBLV was used in indirect ELISA. The primers CBLV3900F and CBLV4576R were used in RT-PCR amplification. Cucumber samples, ‘2106-1’ to ‘2106-5’, collected from Xizhou, Changhua in June 2020 were used for CBLV detection. Sample ‘2106-2’ is the source of CBLV-TW. CBLV-TW-infected and healthy *Chenopodium quinoa* leaf tissues were used as positive and negative controls, respectively. The expected size of amplicons in RT-PCR assay is indicated by an arrow

RAs-CBLV was used to detect CBLV-TW in the field cucumber sample ‘2106-2’, the original host of CBLV-TW, in indirect ELISA (Fig. 6a). In addition, the CBLV-specific primer pair CBLV3900F/CBLV4576R was used to amplify the expected 678-bp DNA fragment from total RNAs of ‘2106-2’ (Fig. 6b). The amplicons were sequenced to confirm correctness.

Symptomatic cucumber samples collected from Yunlin, Changhua and Taichung in central Taiwan in 2020–2022 were tested for CBLV, CCYV, MYSV, WSMoV, SLCuPV, PRSV-W and ZYMV. A total of 75 samples were tested, including 28 in Yunlin, 38 in Changhua and 9 in Taichung. Our result showed that MYSV had the highest detection rate of 74.7% (56/75), followed by CCYV (32.0%, 24/75), PRSV-W (10.7%, 8/75), SLCuPV (9.3%, 7/75), CBLV (8.0%, 6/75) and WSMoV (4.0%, 3/75) (Table 2). Mix-infections of the assayed viruses could be detected. ZYMV was not detected in all tested cucumber samples.

**Table 2:** 2020–2022 cucumber virus detection results in central Taiwan

Location	Collection date	Sample no.	MYSV	WSMoV	CCYV	CBLV	SLCuPV	PRSV-W	ZYMV
Yunlin	2020/01/20	7	7	0	7	0	6	0	0
	2020/11/13	3	3	0	2	0	0	0	0
	2020/12/04	8	7	3	5	4	1	0	0
	2021/07/01	2	2	0	0	0	0	0	0
	2022/06/27	8	8	0	1	0	0	0	0
Changhua	2020/06/22	5	5	0	0	2	0	0	0
	2020/07/07	5	0	0	2	0	0	0	0
	2020/07/24	7	6	0	0	0	0	0	0
	2021/02/04	21	11	0	7	0	0	4	0
Taichung	2021/11/27	9	7	0	0	0	0	4	0
	Sum	75	56 (74.7%)	3 (4.0%)	24 (32.0%)	6 (8.0%)	7 (9.3%)	8 (10.7%)	0

## Discussion

CBLV was first reported in Bulgaria in 2003 and identified as a new member of the genus *Tombusvirus* in the family *Tombusviridae* according to the virion morphology, genome sequence homology and serological relatedness [31]. Ten years later, CBLV was found again in Iran [32]. This is the third report of CBLV emerging in Taiwan in 2020. All CBLV isolates were from cucumber. Similar to co-infection of CBLV with watermelon mosaic virus (WMV) reported in Bulgaria [31], co-infection of CBLV and MYSV has occurred in Taiwan. Nevertheless, different from the previous reports [31, 32], in our study, the pure CBLV cultures, CX-2 and CX-5, were obtained from single lesions on *C. quinoa* leaves. We found that *C. quinoa* is an ideal host to provide CBLV material for virion examination and purification of dsRNA, total RNA and CP due to the consistent viral accumulation 3 days after inoculation. The possibility of contamination by other pathogens, such as MYSV, can also be ruled out.

On the other hand, nanopore sequencing using MinION was performed to identify CBLV. The ONT nanopore sequencing and real-time analysis technology significantly facilitated virus identification within 48 h, but the accuracy of base calling remained relatively lower, with 83.0-95.7% identities to the reference sequence. Also, too many gaps make the reads impossible to assemble. Therefore, Sanger sequencing is necessary to determine the viral whole genome sequence. After validation of the CBLV-TW genome sequence, whole genome sequence analysis of the three CBLV isolates revealed that the current CBLV isolates share the same genome structure and 97-98% sequence homology. However, the genome length of the Taiwan isolate CBLV-TW is one base longer in which the 3′ untranslatable region than that of the other two. CBLV-TW is also phylogenetically distinct from the Bulgarian and Iran isolates (Fig. 3), suggesting that CBLV has diversified geographically.

Furthermore, detection methods for CBLV have been developed and applied in field surveys. In addition to specific primers for RT-PCR analysis, we also produced rabbit antisera against CBLV-TW CP for virus detection. A modified organic solvent-free centrifugation method [26] was conducted to efficiently purify the immunogen. The produced RAs-CBLV is highly sensitive and species-specific, and its utility in indirect ELISA for CBLV detection is comparable to RT-PCR analysis (Fig. 6).

The current incidence of cucumber viruses in Taiwan was investigated. Symptomatic cucumber samples were collected from Yunlin, Changhua and Taichung, the important cucumber cultivation areas in central Taiwan, in 2020–2022 for assay. In addition to CBLV, multiplex-RT-PCR previously developed in our laboratory was conducted to simultaneously detect four important cucurbit-infecting viruses in Taiwan, namely the whitefly-transmitted CCYV and SLCuPV and the thrips-transmitted MYSV and WSMoV. Indirect ELISA with individual antisera specific to CBLV, MYSV, WSMoV, PRSV-W and ZYMV was also performed. Our results showed that MYSV and CCYV were the main cucumber viruses in Taiwan with detection rate of 74.7% and 32.0%, respectively. PRSV-W (10.7%), SLCuPV (9.3%) and CBLV (8.0%) occurred occasionally. ZYMV was not detected in all cucumber samples tested. Mix-infections of MYSV with the other viruses, including CBLV, could be detected, but were not frequently.

Cucumber crops are grown in greenhouses in Taiwan to isolate crops from pests; however, it is difficult to prevent the entry of tiny insects, such as thrips and whiteflies [33]. Melon thrips (*Thrips palmi* Karny), the vector of MYSV and WSMoV [9], and silverleaf whitefly (*Bemisia argentifolii* Bellows & Perring), the vector of CCYV and SLCuPV [4], are common insect pests in greenhouse and have serious effects on many vegetable crops in Taiwan, especially Cucurbitaceae and Solanaceae crops. Although these insect-borne viruses can infect a variety of cucurbit crops, they exhibit different host preferences. Genetic diversity of crop varieties is also associated with virus susceptibility. For instance, both MYSV and WSMoV can infect melons and watermelon; however, MYSV prefers melons, whereas WSMoV prefers watermelons [9]. Our results show that cucumbers are very susceptible to MYSV and CCYV, but resistant to ZYMV. This may be related to the genetic resources of favored cucumber varieties in Taiwan.

No single CBLV infection was detected. Plants infected with CBLV alone may be ignored due to latent infection [31]. Unusual severe symptoms were observed on the cucumber plants co-infected with MYSV and CCYV (Fig. 1a), suggesting a synergy between MYSV and CCYV. The synergistic effect of MYSV and CBLV on cucumber plants can be investigated in the future.

## Conclusion

The emerging CBLV-TW was identified by single lesion isolation and nanopore sequencing. Whole genome sequence analysis revealed that CBLV-TW is closely related, but phylogenetically distinct, to two known CBLV isolates in Bulgaria and Iran. Detection methods including RT-PCR and indirect ELISA have been developed to detect CBLV and to investigate cucumber viruses in central Taiwan. The 2020–2022 field survey results showed that MYSV and CCYV were the main threats to cucumbers, with CBLV, SLCuPV and WSMoV were occasionally occurring.

## Supplementary Information

**Additional file 1: Fig. S1.** Workflow for virus identification and detection in this study.

**Additional file 2: Table S1. **Nucleotide sequences of the primers used in CBLV genome sequencing.

**Additional file 3: Table S2.** Accession numbers of the tombusviruses used for sequence analysis in thisstudy.

**Additional file 4: Table S3.** Nucleotide (nt) identities of the classified reads of CX-2 compared with the genomesequence of cucumber Bulgarian latent virus (CBLV) (acc. no. AY163842).

## Data Availability

The datasets used and/or analyzed during the current study available from the corresponding author on reasonable request.
