# Supplementary figures and images for: Identification of an emerging cucumber virus in Taiwan using Oxford nanopore sequencing technology
Source: Plant Methods. 2022 Dec 22;18:143. doi: 10.1186/s13007-022-00976-x (PMC9773502; doi:10.1186/s13007-022-00976-x)

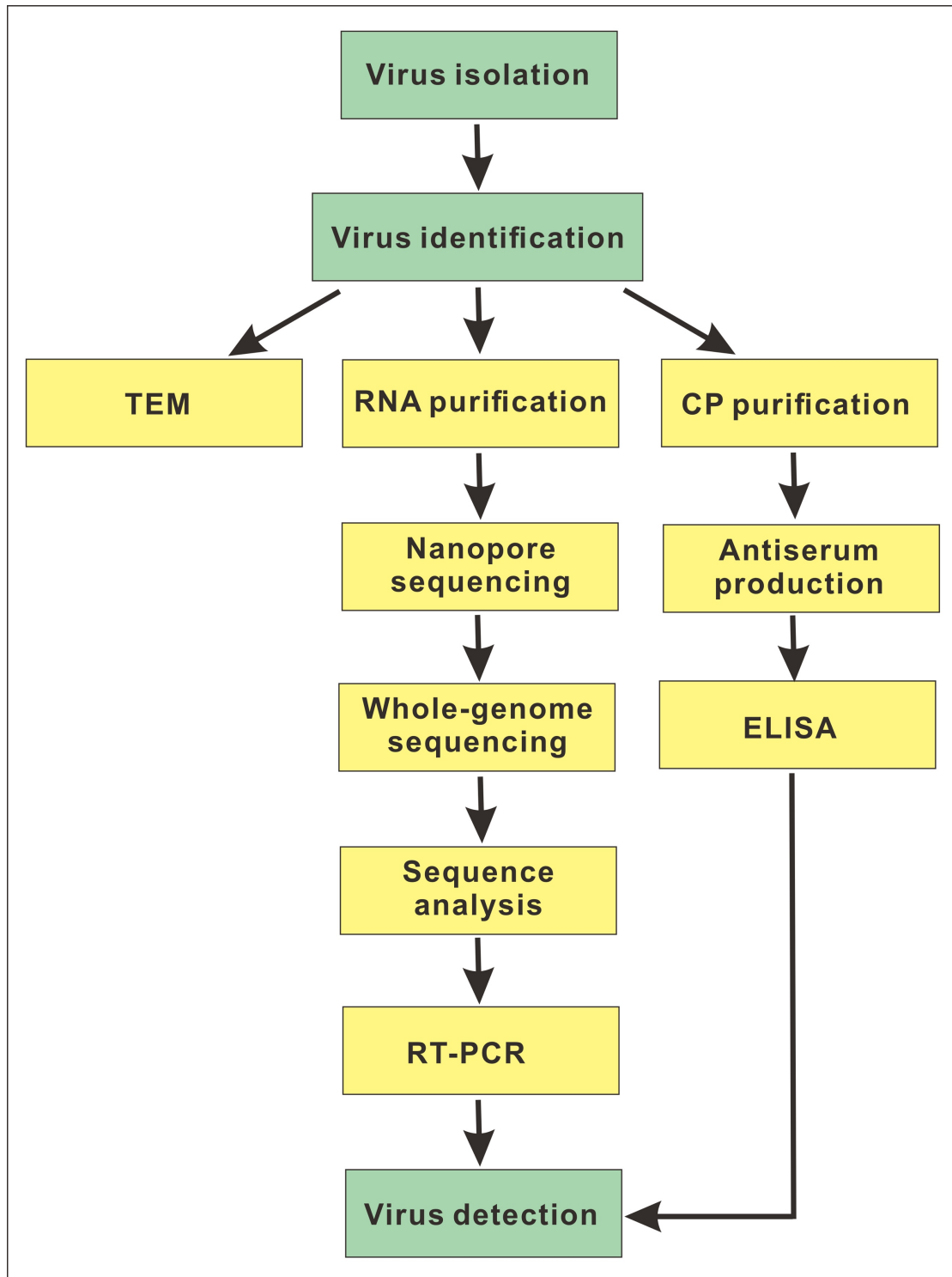

**Fig. S1.** Workflow for virus identification and detection in this study.

Supplement: Supplementary file 1 — Additional file 1: Fig. S1. Workflow for virus identification and detection in this study. [file 13007_2022_976_MOESM1_ESM.pdf]
