# Supplementary material for: Identification of an emerging cucumber virus in Taiwan using Oxford nanopore sequencing technology
Source: Plant Methods. 2022 Dec 22;18:143. doi: 10.1186/s13007-022-00976-x (PMC9773502; doi:10.1186/s13007-022-00976-x)
Supplement: Supplementary file 2 — Additional file 2: Table S1. Nucleotide sequences of the primers used in CBLV genome sequencing. [file 13007_2022_976_MOESM2_ESM.pdf]

Table S1. Nucleotide sequences of the primers used in CBLV genome sequencing

**Primer name**	**Sequence (5′ → 3′)**	**Reference**
CBLV1F	AGAAATTCTCCAGCCTGACCAG	This study
CBLV1888R	CGCATGAAACCAAAGACCATAG	This study
CBLV1818F	ATGAGTGGGGATATAAACACCTC	This study
CBLV3955R	CTCTTAAATACAACTTTCCGA	This study
CBLV3900F	AAAGATTATCCCCTTGGTTTCA	This study
CBLV4576R	GGGCTGCCTTTCGGCAATGTTC	This study
CBLV337R	ATAAACCAATCCCACTTAGCAG	This study
CBLV567R	CAGTAGCTTCCTTCTCATCCTC	This study
CBLV3981F	ACTTCACTGCACCGAGTTACTG	This study
CBLV4284F	GTTAAGGAGTGGTACCCGGATT	This study
Poly G	GGGGGGGGGGAAAGGG	[24]
Poly C	CCCTTTCCCCCCCCCC	[24]
