# Supplementary material for: Identification of an emerging cucumber virus in Taiwan using Oxford nanopore sequencing technology
Source: Plant Methods. 2022 Dec 22;18:143. doi: 10.1186/s13007-022-00976-x (PMC9773502; doi:10.1186/s13007-022-00976-x)
Supplement: Supplementary file 3 — Additional file 3: Table S2. Accession numbers of the tombusviruses used for sequence analysis in thisstudy. [file 13007_2022_976_MOESM3_ESM.pdf]

Table S2. Accession numbers of the tombusviruses used for sequence analysis in this study

Virus name	Abbreviation	Acc. no.
Cucumber Bulgarian latent virus	CBLV-TW	MW359100
Cucumber Bulgarian latent virus	CBLV-W12-101	KJ572966
Cucumber Bulgarian latent virus	CBLV-Bulgaria	AY163842
Artichoke mottled crinkle virus	AMCV	NC_001339
Carnation Italian ringspot virus	CIRV	NC_003500
Cucumber necrosis virus	CNV	NC_001469
Cymbidium ringspot virus	CymRSV	NC_003532
Eggplant mottled crinkle virus	EMCV	NC_023339
Grapevine Algerian latent virus	GALV	NC_011535
Moroccan pepper virus	MPV	NC_020073
Pelargonium leaf curl virus	PLCV	NC_030452
Pelargonium necrotic spot virus	PNSV	NC_005285
Tomato bushy stunt virus	TBSV	NC_001554
Havel river virus	HaRV	NC_038690
Limonium flower distortion virus	LFDV	NC_038691
Neckar river virus	NRV	NC_038927
Petunia asteroid mosaic virus	PAMV	NC_038692
Sikte waterborne virus	SWV	NC_038693
