# Supplementary material for: Identification of an emerging cucumber virus in Taiwan using Oxford nanopore sequencing technology
Source: Plant Methods. 2022 Dec 22;18:143. doi: 10.1186/s13007-022-00976-x (PMC9773502; doi:10.1186/s13007-022-00976-x)
Supplement: Supplementary file 4 — Additional file 4: Table S3. Nucleotide (nt) identities of the classified reads of CX-2 compared with the genomesequence of cucumber Bulgarian latent virus (CBLV) (acc. no. AY163842). [file 13007_2022_976_MOESM4_ESM.pdf]

Table S3. Nucleotide (nt) identities of the classified reads of CX-2 compared with the genome sequence of cucumber Bulgarian latent virus (CBLV) (acc. no. AY163842)

Reads	Sequence	Length (nt)	Corresponding position	Identity (%)
1	GTAGTGACGCCGTTCAGTTACGTATTGCTGAGC<br>AACAGATTTTGTTGGACAGCGTGCGATTGGTGT<br>AGAAACTGGACCCAAGATTGACATGTTCTCTCT<br>CATATGTAAAGTGGTCCTCAGGTATATGCAGAC<br>AGGAAAATAGAGCACAAGGTCGATGGACTGAG<br>TGGATTTGTGGTTGAGTTACTCAAGACTGATGC<br>CGCTGCTAAGTGGGATTGGTTTATGAGGAGGA<br>GCGAGGAGAACTGACTACGTTGGTGCGGTCAG<br>TTGTCTTTCTGCCATAGCCATCCTTCCACTCTGG<br>TGCGCCAAGTACTCTCGGCTGGCAGTGGAATGT<br>TGTTAACACCAGGTTTATTGTTGCAGCAACATG<br>CGCTACCCTACCCAGGGAAGGGCGATAACAAC<br>ATCGAAACTTGAGGGTAGAGGCCAGGGAACAC<br>ATGGAGGATGAAGGAAGCTACGATGCTTAGTG<br>GATTTGGGAAGCAAGCGGCTCGAGAAGAGAGG<br>ATGA	493	168-610	89.16
2	ATTGTATGTTCAGTTTGTTGCTATACGGATTCGT<br>CTCACAGTGATGTACCCTCCACTCATCCCTATT<br>CGGACTTTCGTCAGAAGGCGGAAGGGAGTAAT<br>GGTACTTCCATATCTTCCATCCCAACATTCACC<br>AACAGCTTGCTTCCCAGTGTGATCTCCTC	161	3758-3897	95.71
3	ATTGTACTTCGTTCAGTTACATTGCTGGATCGA<br>TGCTATCACATAGGACCTGTAAAACAACCTCCC<br>ACGAGGAAGTGTTTCGCTTAGATATTACGCCTG<br>TTGCTACCTCGAATCTGTCAGGAAATGGACCAC<br>TGTCTCCGAGTCCTTATCAAAGAATAGCCCAAC<br>TCTACCAAACTCCGTGAGGTCGCACACAGGGA<br>CTGTGGTGGAAAGATAATCGACTGGTGAACTT<br>ATGTTTTGTCGAAATTGGATGCTATATTAAGCA<br>ACCAGAGGAAACATTCACCGCGTTCGATGGGG<br>TTGGACGCGGACTATGAATACAGCAGCTT	323	2886-3148	83.03
4	GATGTACTTCGTTCAGTTACGTATTGCTACCCT<br>CCATTTATGGACATTTGTGACGGTGTCTATTCG<br>CTGGTGCTTCCTAGGCGGATCATAGAGCTTGAA<br>TCTAGAGTAGAACTTGTCCACCACTGGTATACC	1025	1674-2325	93.58

	AGCAGACAAAACACGCCCACATGTTGTGCATT<br>ACTCCACGCGCGCCTAGTTTGCTCATCTTTAAT<br>ATTATTAACACAATGAGAATCCTTAGACATTGC<br>CGTCCTTACATTTCGGACCATCTTCCAACCGCC<br>CTGAAAGTTGACTGGATGAGCCTGACAAAATT<br>CAATCTCTTCCAATTGAAATACGAGTTCCTCTA<br>CCTTCATGGTGTACCCCAAATTGAGAAATACCC<br>AGGTAGTTTCGCCTGTACCTGTTTAAGACACCG<br>CTTCTCAATAATGAAACACAGTCATCTCCACAG<br>TTTGCCAGACTATATTCATTGATGCCACAGTAT<br>CGCATGAAACCAAAATTCATAGAGCACATTAA<br>CAAATAATTTCCCAGAGGTGTTTATATCCCCCT<br>CATTCTGCAACCCTCCTTTCGATACGAGATAGT<br>GCCATCAGGCACAACATCCCTTCCTTATTATGG<br>AGTTGCCATTCCAATAGTTGGTCTAAGACCTTA<br>CTACCCGGGTAAAGGGCCCGGTAGAAGCCATG<br>TTCAAACCGTACAGGCGAAGCTACCAGGTATTT<br>CCTTAATTTGGGGTACACCATGAAGGTAGAAG<br>ACAATGTTTCAATTGGAAGAGATTGAATTTTGT<br>CAGGCTGTCAGTCAACTTTCAGGGCGGTTGGAA<br>GATGGTCCGAAATGTAAGGACGGCAATGTCTA<br>AGGATTCTCATTGTGTTAATAATATTAAAGATG<br>AGCAAACTAGGCGCGCGTGGAGTAATGCACAA<br>CATGTGGGAGGTAGAGCTTTGTCTGCTGGTATA<br>CCAGTGGTGGACAAGTTCTACTCTAGATTCAAA<br>AATATGATCCGCTAAGCACCAGCGAATAGACA<br>CCGTCACAAATGTCCATAAATGGAGGGTAGCA<br>ATACGTAACTT			
5	AGTGTACTTCGTTCAGTTACGTATTGCTGATGC<br>CGCATAGTTACCGTATCTCCCGACCGAATCCAA<br>TAACTCCCGGTGCAGTGAAGTTTCTTTCAGTCG<br>TATCTGTAATATCTCTTAAATACAACTTTCCCG<br>AAACCCAGCTTTCCTTGAAACCAAGGGGATAA<br>TCTTTATACGGATTCGTCTCTGATGATGTACCTC<br>CACTCATCCTATTCGGCGGACTTTCGTCAGGAA<br>GTTGGAAGGGGGTAATGGTACTATATCTTCCAT<br>CCCAACATTCACCAACAGCTTGTTTCCCAGTGT<br>GATCTCCTTGTACAATTCGTTCCATGGTTTGTTA	2337	1719-4039	90.18

	ACTTGTTTATTACTCAGTATCCATTGAAATGAG<br>TCTGTTCCAAGAGGCCCTAAAGTAACGTGCATT<br>TCAGCCGCTGATAATATCGCGTGTGGTGCGCAC<br>AGCAAAGAAATTCCAAAACGTAGACGACGCAG<br>CAGTGAAATTAAAGTTAAGATTATGACCTGGTG<br>TAGATGCGATGACTTGACAAATATAAATGATCT<br>TTGTGGCGGAAACCACCACACTTTGTCCGGTAA<br>TTGTCAAAGCACTAGGATTAGATGTCAGATTTC<br>CCCGCCGTCATTTTGCAGTTCCATCAAGATGAG<br>TGACAAGATAGGTGCCAGCTATAGTAGGTGTA<br>AATGCTGCCAATGTTGCACCGAAGGACGCCAA<br>GTTAAAATAACTGGGCGGTGTGAAGTATTTAAC<br>TGAATTACCAGCATTACCTGTTATCTCCTCCAT<br>GGTATTTGAGGATGGTTGGGGGTTCGCGGGCTC<br>CACGGTGTATTGCACAAAGATATCACCATATGC<br>ATTGCTCGTGCTCCCCCCATAAACCACGTAACC<br>AAAACCGACCCAAATCAATGAGTTTGAGTGTC<br>AGTTACAGTACTATCACTAATAAACCACGATCG<br>ATGCTATCACATGGGACCGCCAAACAACTCCCC<br>CGAGGAGGTGTTTCAGCAAGATGTGCCATATTT<br>GCTACCTCAGATCTGTCAAATGGGCCACTGTCC<br>TCCGAGTCCTTATCAAAGAATAGCCCAACTCTA<br>CCAACATCCGTGGTCGCACACATAGGAACGTA<br>GTGGAAAAGATAAATCCTGAGGTGAACTTATA<br>CTTGTCGAAAGTGGATGCTATATTAAGCAACCG<br>AAACACTGCCGCGTTCGTGGGGTTGACGCATTA<br>AGACCTGGCCCAAGTCCGGTTCAACTGAAAAG<br>TACCATCATTAACACCATTAACTTTTGTGGCTG<br>ACATACTCACGATGGGTGATGTTGACACCTCCG<br>TGTTCGCATAAACTTAGGTCTATAGCCCTCAGC<br>TTTGCCTGGAGGTGGCCACAGGGGAGCCACAA<br>TAGATCAGGTGATGCTCCTACTACATTGGACAC<br>AATTCCATTCTTCTTCTTCACCCTCTTGGAAACT<br>TTGGCGTAAATCAATTCAGGCCATCATATATAA<br>TCTTCCTGTTATTATAGACTGCTGTTGCGCCTGC<br>TTTGGCCGCGGGGCCAACATAAGGCACAATGT<br>TCCCTGCTTTCCTAAGCACTAAAGCCATGTTTT<br>CAATGTTGTTTGGTCAAGCTGCGGCAGAGTCGA			

	GGGTCTCGGCTCGCGCGAGTCGGTACCCTCTTT<br>AAAGAGATCATGTCCCATCGGTCCAATCTGTCC<br>TCCAGAGCTATCTGTTCATCCCCGATAGCTTAA<br>ATGCTTGCCAGAAACTAACCCTACTTTCTGGTG<br>TGGATACGTCTGCAGAACCCCTCCATTTATGGA<br>CATTTGTGACGGTGTCTATTCGCTGGTGCTTCC<br>CTAGGCGGATCATAGAGCTTGAATCTAGAGTA<br>GAACTTGTCCACCACTGAAGGTATACCAGCAG<br>ACAAAGCTCTACCTCCCACATGTTGTGCATTAC<br>TCCACGCGCGCCTAGTTTGCTCATCTTTAATATT<br>AACACAATGAGAATCGCCGAACATTTACAATC<br>CTTACATTTCGGACCATCTTCCAACCGCCCTGA<br>AAGTTGACTGGATGAGCCTGACAAATTCAATCT<br>CTTCCAATTGAAATACGGGTTCTCTACCTTCAT<br>GGTGTACCCCAAATTAAGGAAATACCCAGGTA<br>GTTTCGCCTGTACCTGTTTAAGACACCGCTTCT<br>CAATAATGAGAACACAGTCATCTCCACAGTTTG<br>CCAGACTATATTCATTGATACCACAGTATCGCA<br>TGAAACCAAAGACCATAGAGCACATTAACAAA<br>TAATTTCCCAGAGAGGTGTTTATATCCCCACTC<br>ATTCTTAAACCCCTCCTTTCGATACGAGGGAAT<br>AGTGCCATCAGGCTGCTTATCCTTCCCCTTATT<br>ATGGGTGCCATTCCAATAGTTGGTCTAGACCTT<br>ACTG			
6	ATTGTACTTCGTTCGGTGCAGCATTATCTTCTGT<br>GTGGAAGACACCCGCAAGACCGCGCTATTCAG<br>GTTTCTCAGGCAGTGATTATGGACAAAGCCTGA<br>GCGCGGTCTGTGGAAAATCTTCTGTGTGAAAGG<br>AGAACAGTTCGCACTCCACAACCCAAACCTGG<br>GCCTTTGCTAGTCTTTCCCCGTTCAGGAAGCTG<br>TCTGTGAGAAAGGTCGGAATTTTAGCAGCAGG<br>GTATGGCGGATTTCTGTCATACTACAGTGGAGC<br>GAAATTACATTGCCTCGCGGGCGGTGGAGAGT<br>CTGCGCTATCACTCCTGTCTGTGAGCGTGACAG<br>TCATTTGACTACTTTTTGTAAAGGCAGAGAAGA<br>TATCGACTAATGGGTGATCCTGCACCTAGGGTT<br>ATTCAGCCTCGCAACCCTGAGTACAATGTTGAA<br>TTAGGTCGTTTTCTGCGGCGACAGGAGGTAACT	641	1127-1686	86.55

	CACAGCGGTTGACGCAGTTTTTTGGGTGAAACC ATGTATCAAGGGGTACACTGCAGATGAGGTCG ACGCTATCTTCAGGGACAAATGGGACAGGTTT GATATTTATGTGCCGTCTGGCTGATGCGTCGAG GTTTGATCAACACTGTTCCGTAGGAGCCACGGT TTGTTTGACAGCTCACCAG			
7	TTGTACTTCACGTTCAGTTACGTATTGCTCCTCC ATTTATGGACATTTGTGACAGTGTCTATTCGCT GGTGCTTCCTAGGCGGATCATAGAGCTTGAATC TAGAGTAGAACTTGTCCACCACTGGTATACCAG CAGACAAAGCTCTACCTCCCACATGTTGTGCAT TACTCCACGCGCCTAGTTTGCTCATCTTTAATAT TATTAACACAATGAGAATCCTTAGACATTGCCG TCCTTACATTTCGGACCATCTTCCAACCGCCCT GAAAGTTGACTGGATGAGCCTGACAAAATTCA ATCTCTTCCAATTGAAATACGAGTTCCTCTACC TTCATGGTGTACCCCAAATTGAAATACCCAGGT AGTTTCGCCTGTACCTGTTTAAGACACCGCTTC TCAATAATGAGAACACAGTCATCTCCACAGTTT GCCAGACTATATTCATTGGTACCACATTATCGC ATGAAACCAAAAAGACCATAGAGCACATTAAC AAATAATTTCCAGAGAGAGTGTTATATCCCCCT CATTTCGTGCAACCCTCCTTTCGATACGAGATA GTGCCATCAGAGCACATGTCCTTCCCCTTATTA TGGGTGCCATTCCAATAGTTGGTCTAAGCCTTA CTACCCGGGTAAGGAGCCCATTAGAAGCCACA TGTTCAAACCGTAAGGCTTCCACGGAACAGTGT TGATCAAACCTCGACGCATCTAGGCCAATGGC ACATGGCTTATCAAACCTGTCCCATTTGTCCCT GAAGATTAGCGCCGACCTCATCTGCCCAGTGTA CCCCTTGATACATGTGGTTTCACCAAAAACTGC GTCAACCGCTTTCATGAGCTTACTCTCCAAATG CCGCAGAAAACGACCTAATTCAACATTGTACTA GAGGTTGCGAGGCTGAATAACCCTAGGTGCAG GATCACCCTTACTAGTCGATATCTTCTCTGCCTT TACAAAAGTAGTCAAATGACTGTCCACGCTCAC AGACAGGAAGATGATATGAACTCTCACCGCCC GCGGTAGAT	1029	1330-2324	93.17

8	TTGTACTTCGTTCAGTTACGTATTGCTAGCGTG<br>GAGTAATGCACAACATGTGGGAGGTAGAGCTT<br>TGTCTGCTGGTATACCAGTGGTGGACAAGTTCT<br>CTGGAGTCAAACTCTATGATCCATACTAGGAAG<br>CACCAGCGAATAGACACCGTCACAAAATGTCC<br>ATAAATGGAGGGTTCTGGTGGTGATTATGTAAT<br>CACACCAGAAAGTAGGGCTAGTTTCTGGCAAG<br>CATTTAAGCTTACCGGGGATGAACAGATAGCC<br>CTTGAGGACAGATTGGACCGATGGGACATGGA<br>TCTCTTTAGGGGTGAGGGTACCGACTCACATGG<br>GCGAGTATCCTCGACTCTGCAACTTGACCAAAC<br>AACATTGAAAACATGGCTTTAGTGCTTAGGAA<br>AGCAGGGAACATTGTGCCTTATGTTGGCCCCGC<br>GGCCAAAGCAGGCGCAGCAGCAGTCTATAATA<br>ACAGGAAAGAGCAATACGTAACTT	479	2178-2617	92.99
9	AGTGTACTTCGTTCCAGGTTACGTATTGCTGTTT<br>GAGTCAAGCTGCGGCAGAGTCGAGGATACTCG<br>GCTCATGTGAGTCGGTACCCTCACCCCAAAGAG<br>ATCCATGTCCCATCGGTCCCAATCTGTCCTCAA<br>GGGCTATCTGTTCATCCCCGGTAAGCTTAAATG<br>CTTGCCAAACTAACCCTACTTTCTGGTGTGATT<br>ACATAATCACCACCAGAACCCCTCCATTTATGG<br>ACATTTGTGACAGTGTCTATTCGTAGTGCTTCC<br>TAGGCGGATCATAGAGCTTGAATCTAGAGTAG<br>AACTTGTCCACCACTGGTATACCAGCAGACAA<br>AGCTACCTCCCACATGTTGTGCATTACTCCACG<br>CGCGCCTAGTTTGCTCATCTTTAATATTATTAAC<br>ACAATGAGAATCCTTAGACATTGCCGTCCTTAG<br>CAATACATGT	438	2113-2517	93.37
10	GTGTACTTCGTTCAGTTGTATTGCTACCGGTAA<br>TTGTCAAGCACTAGGATTAGATGTCGAGATGTT<br>CCCCGCCGTCATTGCAGTTCCATCCAAGATGAG<br>AGTGACAAGATAGGTGCCAGCTATAGTAGGTG<br>TAAATGCTGCCAATGTTGCACCGAAGGACATAT<br>CAAGTTAAATAACTGGGCGGTGTAGTGACAAC<br>TGAATTACCAGCATTACCTGTTATCTCCTCCAT<br>GGTATTTGAGGATGGTTGGGGTTCGCAGAGCTC<br>CACGGTGTATTGCACAAAGATATCACCATATGC	2035	2348-3508	91.78

	ATTGCTGGTGCTCCCCATAAACCACGTAACCAA<br>ACCGACCCAAATCAATGGATTTGGTGTCAGTTA<br>CGGTACTATCATAATAAACCGATCGATGCTATC<br>ACATGGGACCTGTAAAACAACCTCCCCAAGGA<br>GGTGTTTCAGCAAAGATGTGCCATATTTGCTCA<br>GATCTGTCAAATGGGCCACTGTCCTCCGAGTCC<br>TTATCAAAGAATAGCCCAACTCTACCAACCTCC<br>GTGGTCGCACATAGGAACGTAGTGGAAAGATA<br>ATCTGGTGAACTTATACTTGTCGAAATTGGATG<br>CTATATTAAGCAACCAGGGAAACACTGCCGCG<br>TTCGTGGAGTTGACGCGATGGCACCTGGCCCAA<br>AGTCGGTTCAACTGAAAAGTACCATCATTAACA<br>CCATTAACTTGTGTGACATACTCACGATGGGTG<br>ATGTTGTACCTCCACGTGTTCGCATAAACTTAG<br>GTCTATTAGCCCTCAACTGCCTGGAGAGTGTAC<br>AGGGAACCACAATAGATCAGGTGATGCTCTAC<br>TACATTGGACACAATTCCATTCTTCTTCTTCACC<br>CTCTTGAAACTTTGGCGTAAATCCGAATTCAGG<br>CCATCATATATAATCTTCCTGTTATTATAGACT<br>GCTGTTGCGCCTGCTTTGGCCGCAGGGCCAACA<br>TAAGGCACAATGTTCCTGCTTTCCTAAGCACCT<br>AAAGCCATGTTTTCAATGTTGTTTGGTCAAGCT<br>GCGGCAGAGTCGAGGATACTCAGCTCATGTGA<br>TCGGTACCCTCACCCCCAAAGAGATCCATGTCC<br>CATCGGTCCAATCTGTCCTCAAGGGCTATCTGT<br>TCGTGTCGGTAAGCTTAAGCGCGCGAAGTAAC<br>CTACTTTCTGGTGTGGGGTACCATTTCCACATG<br>AGCCGATATCCTCGACTCTGCCTGTGACCAAAC<br>AGCATTGAAAGCATGGCTTCCGAATTCTTAGAA<br>AACAAGTAGTATATGTGGCCCACTTGGCAAAA<br>GCAGACCTCAACAACAGTCTATAATAACAGGA<br>TTATATATGATGGCTGAGTGGATTTACCCATAA<br>AGTTTCAAGAAAGTGAGAAATAATTATGTCAA<br>GTGCTTAATGGATCTGTGTGGCCTGTAACCACA<br>CCTCCGACAGTAATAGATAAGTTTATGCGGCAG<br>AACACGTGGAAATCAACATCACCATCGTTCTAG<br>GCCAGGCGGGTATCACGGCAGTGTCCTGGTTGC<br>TTAATATAGCAATTTCGGCAAATTATAAGTTCA			

	CCAGTTATCTTTTCCCGCACGTTCCTATATTATT<br>AAAACTACAGAGTTGAGTAGGATTGGGCTATT<br>CTTTGATAAGGACTCGGACGAGTGTACATTGAC<br>TATCTGGTGTAAATATGGCAATCTTGCTGGTAC<br>ACTCCTTGGGAGGAAGGGATTGTTTTTACGGGG<br>GTCCCCATGTGATGCATCGATCGGTTTTATTAA<br>TTGATAGTACCGTAACTGACACCAAATTGATTT<br>GGGTCGGTTTCAGTTACGTGGTTTATGGGAAGT<br>ACCAGCACCTATGGTGATAATCTTGTGCAATAC<br>ACCGTGAATACTAAACTAGCCAGCCTCAGAAT<br>ACCATGGAAGGAGATAACAGGTAATGCTGTGT<br>CAGTTGTCTACACCGCCCAGTTGATCGCTTCCG<br>GTACTGTACAGCTACACCCTGCATAGCAGCACC<br>TGTGTCACTCATCTTGGATAGACAAAATTCGAC<br>ATCTATCTAGTGCTACGACAATTACCGGTACCC<br>G			
11	AACGTACTTCGTTCAGTTACGTATTGCTGGTGT<br>AGATCGATGACTTGACAAATATAAATGATCTTT<br>GTGGCGGAAACCACCACACTTTGTCCGGTAATT<br>GTCAAGCACTAGGATTAGATGTCGAGATGTTCC<br>CCGCCGTCATTGCAGTTCCATCAAGATGAGAGT<br>GACAAGATAGGTGCCAGCTATAGTAGGTGTAA<br>ATGCTGCCAATGTTGCACCGAAGAGCCGCTAA<br>GTTAAAATAACTGGGCGGTGTAGTGACAACTG<br>AATTACCAGCATTACCTGTTATCTCCTCCATGG<br>TATTTGAGGATGGTTGGGGTTCGCGGAGCTCCG<br>GTGTATTGCACAAAGATATCACCATATGCATTG<br>CTCATTACTCCCCACCAAAACCACGTAACCAAA<br>CCGACCCAAATCAATGAGTTTGGTGTCAGTTAC<br>GGTACTATCACTAATAAACCGATCGATGCTATC<br>ACATGGACCTGTAAACAACCTCCCCCCAAGGA<br>GGTGTTTCAGCAAGATGTGCCATATTTGCTACC<br>TCGAATCTGTCAAATGGGCCTATCCTCGAGTCA<br>TATCAAAGAATAGCCCAACTCTACCAACCTCTG<br>TGGTCGCACATAGGAACGTAGTGGAAAGATAA<br>TCTGGTGAACTTATACTTGTCCGAAATTGGATG<br>CTATAAGTAAGCAGCAAAACCTGCCGCGTTCGT<br>GGGGTTGACGCGATAGGCACCTGGCCCAAAAG	1409	2728-3573	90.64

	TCGGTTCAACTGAAAAGTACCATCGGCATTAAC<br>ACCATTAACGTGGCTGACATACTCACGATGGGT<br>GATGTTGACACCTCCACGTGTTCGCATAAGAAG<br>CTTAGGTCTATTAGCCCTCAACTGCCTGGAGGT<br>GGCCACAGGGGTTGGTAGAGTTGGGCTATTCTT<br>TGATAAGGACCGGAGATAGTGGCCCATTTGAC<br>AGATCTGAGGTAGCAAATATGGCACATCTTGCT<br>GAAACACCTCCTTGGGGGAGTTTGTTTACAGGT<br>CCATGTGATAGCATCGATCGGTTTATTAGTGAT<br>AGTACCGTAACTGACACCAAACTCATTGATTTG<br>GGTCGGTTTGGTTACGTGGTTTATGGGGGAGCA<br>CGAGCAATGCATATGGTGATATCTTTGTGCAAT<br>ACACCGTGAGCTCCGCGAATTTAACCATCCTCA<br>AATGCCATGGAGGAGATAACAGGTGTCACTGG<br>TAATTCAGTTCACTACACCGCCCAGTTATTTTA<br>ACTTGGCGTCCTTCGGTGCAACGTGGCAGCATT<br>TACACACCTGCTATAGCTGGCACCTATCTTGTC<br>GCTAAGTGGAGACAATGACGGCGAAGAGCGCG<br>AAATCTCGACATCTATCTATGCTGACAATTACC<br>GGACAAAGTGTGGTTTTCCTGCAAGATCATTTA<br>TATTTGTCAGGTCATCGCATCTACACCAGCAA			
12	AATCGTACTTCGTTCAGTTACGTATGTTGAAGA<br>GTGGTCTATTTCAAGAGGCCCCTAAAGTAACAC<br>ATGGCTAAGCGCTGATAATAATATCGCGTGTGG<br>TGCGCACAGCAAAAGAAATTCCAAAACGTAGC<br>CGACGCAGCAGTGAAATTAAAGTTAAGATTAT<br>GACCTGGTGTAGATGCGATGACTTGACAAATGT<br>AAATGATCTTTGTGGCGAAACCACCACACTTTG<br>TCCGGTAATTGTCAAAGCACTGGATTAGATGTC<br>GAGATGTTCCCGCCGTCATTGCGGTTCCATCCA<br>AGATGAGAGTGACAAGATAGGTGCCAGCTATA<br>GTAGGTGTAAATGCTGCCAATGTTGCACCGAA<br>GGACATGAGATTAAAATAACTGGGCGGTGTAG<br>TGACAACTGAATTACCAGCATTACCTGTTATCT<br>CCTCCATGGTATTTGAGGATGGTTGGGGTTCGC<br>GGAGGCTCGGTGTATTGCACAAAAGATATCAC<br>CATATGCATTGCTCGTGCTCCCCATAAACCACG<br>TAACCAAACCGACCCAAATCAATGAGTTTGGT	1269	3142-3704	90.97

	GTCGGTTACGGTACTATCACTAATAAACCGATC GATGCATATGGTGATATCTTTGTGCAATACACC GTGGAGCTCCGCGAACCCAACCATCCTCAAAT ACCATGGAGGAGATAACAGGTAATGCTGGTAA TTCAGTTGTCACTACGCCCAGTTATTTTAACTTG GCGTCCTTCGGTCACAACATTGGCAGCATTTAC ACCTACTATAGCTGGCACCTATCTTTGTCTCTC ATCTTGGATGGAACTGCAATGACGGCGAGGGA ACATCTCGACATCTAATCGAATAACATTACCGG ACAAAGTGTGGTGGTTTCCTCGCAAAGATCATT TATGTTTGTCAGTCATCCTCGTCTACGTAGTCAT AATCTTAACTTTAATTTTTGCGTCGGCTACGTTT TGGAATTTCTTTTTGCTGTGCGCATCATCGGTA ATGAGTACTTACAACAAAGAAATTCCAAAACG TAGCCGACGCAGCGGTGAAATTTCTTTGCTGTA CGCACCACGCTGATAATAATATCGCGTGTGGTG CGCACAGCAATTCCAAAACGCTTAGCCAAACA TAGCAGTACGAGATAAAGTTAAGATTATGACC TGGTGTAGATCGTGACTTACATAAATATAAATG ATCTTTGTGGCGGAAACCACCACAAAGATCATT TATATTTGTCAAGTCATCACATCCCTACCGAAT CATAATCTTAACTTTAATTTCACTG			
13	AATTACTGATTCAGTTACGTATTGCTCATGGAG GAGATAACAGGTAATGCTGCTATTGGTCGGTGT CACTACACCGCCCAGTTATCTCCTCCCATGGTA TTTGAAGAATGGTTGAGTTCATGAAACTCCGGT GTATTGCACAAAGATATCACCATATGCGTGCTC GTGCTCCCCATAAACCACGTAACTAAACCGACC CAAATCAATGAGTTTAGTATCAGTTACGGCCAC TATCACTAATAAACCGATCGATGCTATCACATG GGACCTGTAAAACAACCTCCCCCCAAGGAGGT GTTTCAGCCAAGATGTGCCATATTTGCTACCTC AGATCTGTCAAATGGGCCCGCATCCTCGAAGTC GCTTATCAAAGAATAGCAACTCTACCAACCTCC GTGGTCGCACACATAGGAACGTAGTGGAAAGA TAATCTGGTGAACTTATACTTATCCGAAATTGG ATGCTATATTAAGCAACCAAGGAAGGCTACTG CACCCGATCGTGGGGTTGACGCGATAAACACC	577	2825-3322	87.80

	TGGCCCAAAGTCCGGTTCAACTGAAAAGTCTA<br>ATCATTGCTTACCATTAACTG			
